# Antimicrobial peptides: from discovery to developmental applications

**DOI:** 10.1128/aem.02115-24

**Published:** 2025-04-03

**Authors:** Qi Zhang

**Affiliations:** 1 Centre for Eye and Vision Research 580408 , Hong Kong, Hong Kong; Centers for Disease Control and Prevention, Atlanta, Georgia, USA

**Keywords:** antimicrobial resistance, antimicrobial peptides, characteristic, identification path, developmental application

## Abstract

Antimicrobial resistance (AMR) has emerged as a significant crisis in global health. Due to their advantageous properties, antimicrobial peptides (AMPs) have garnered considerable attention as a potential alternative therapy to address the AMR crisis. These peptides might disrupt cell membranes or cell walls to exhibit antimicrobial activity, or modulate the immune response to promote recovery from diseases. In recent years, significant progress has been made in the research of AMPs, alongside the emergence of new challenges. This review first systematically summarizes and critically discusses recent advancements in understanding the characteristics and current landscapes of AMPs, as well as their regulatory mechanisms of action and practical applications, particularly those reported or approved within the last 5 years. Additionally, the principles, paths for their identification, and future research trends in AMPs are also analyzed following a discussion of the advantages and disadvantages of AMPs in comparison to conventional antibiotics. Unlike significant prior literature in this field, this report has summarized the latest major discovery methods for AMPs and, more importantly, emphasized their practical applications by supporting various viewpoints using selected examples of AMPs’ applications in real-life scenarios. Besides, some emerging hot topics of AMPs, including those derived from gut microbiota and their potential synergistic effects in combating AMR, were profiled. All of these indicate the originality of the report and provide valuable references for future AMP discoveries and applications.

## INTRODUCTION

As antimicrobial resistance (AMR) worsens, the efficacy of conventional antibiotics is declining (1–4). This has spurred the exploration of new antimicrobial strategies, with antimicrobial peptides (AMPs) emerging as a promising alternative (5, 6). AMPs, typically comprising 10 to 50 amino acids, are naturally occurring molecules found in plants, animals, and microorganisms (6). Typical representative AMPs are nisin (found in the 1920s) and magainin (first isolated peptides from the skin of amphibians in the early 1980s) (7). Thousands of AMPs have since been identified, showcasing diverse structures and antimicrobial activities. Their potential applications in biomedicine, agriculture, and food safety are increasingly recognized (8, 9), making them vital candidates for addressing AMR (10).

AMPs are synthesized via two main pathways: ribosomal and non-ribosomal synthesis (11). Ribosomal synthesis involves gene transcription into mRNA, translation in ribosomes, and post-translational modifications like cleavage or glycosylation. These AMPs, such as defensins, are typically evolutionarily conserved and widespread across species (12–14). In contrast, non-ribosomal synthesis relies on non-ribosomal peptide synthetases (NRPS), which assemble amino acids into peptide chains without mRNA. NRPS enable modular assembly, incorporating diverse amino acids and post-synthetic modifications like cyclization or methylation, enhancing peptide stability and activity. Gramicidin A, a representative of non-ribosomally synthesized AMPs effective against gram-positive bacteria, is produced by *Bacillus brevis*. This AMP disrupts bacterial cell membranes by forming channels that permit the passage of ions, ultimately leading to cell death (15). Another famous non-ribosomal synthesized AMP is colistin (polymyxin E). As a last-resort antibiotic against extensively drug-resistant gram-negative bacteria, this lipopeptide is synthesized via NRPS, which involves cyclization, fatty acid attachment, and post-synthetic modifications, such as hydroxylation and methylation (1).

In this review, the characteristics, sources, and applications of AMPs, along with various principles and methods for discovering and identifying AMPs, are systematically analyzed with the aim of facilitating the effective development of AMPs in the future.

## CLASSIFICATIONS AND CHARACTERISTICS OF AMPs AND THEIR REGULATION AND MECHANISMS OF ACTION

The classifications of AMPs vary, depending on their different characteristics. Notably, these classifications are often not isolated but interconnected, with differences in one characteristic often influencing other aspects. According to their source, antimicrobial spectrum, and mechanisms of action, AMPs can be classified into subsets, in which host defense peptides (HDPs) and bacteriocins are two of the most common subcategories (16). HDPs are produced by a wide range of host cells, including those in humans. As the first line of defense for the host immune system, they exhibit a broad spectrum of antimicrobial activity against bacteria, fungi, viruses, and even some parasites (8). Moreover, these molecules may play critical roles in regulating immune responses and promoting wound healing (17). Consequently, scientists commonly use the term defense peptides to reflect the multifaceted nature of these molecules in contemporary research (18). Correspondingly, as the other subset of AMPs, bacteriocins are produced by bacteria to inhibit the growth of similar or closely related bacterial strains, primarily functioning in inter-bacterial competition (19). Unlike HDPs, bacteriocins are more specialized and often have a narrower spectrum of activity. A typical example is sakacin P, a narrow-spectrum bacteriocin derived from *Lactobacillus sakei*, which targets a limited range of pathogens like *Listeria monocytogenes* (20). This characteristic leads to minimal impact on probiotics in the human gut, thereby enhancing its safety for application in the food industry.

**Fig 1:**
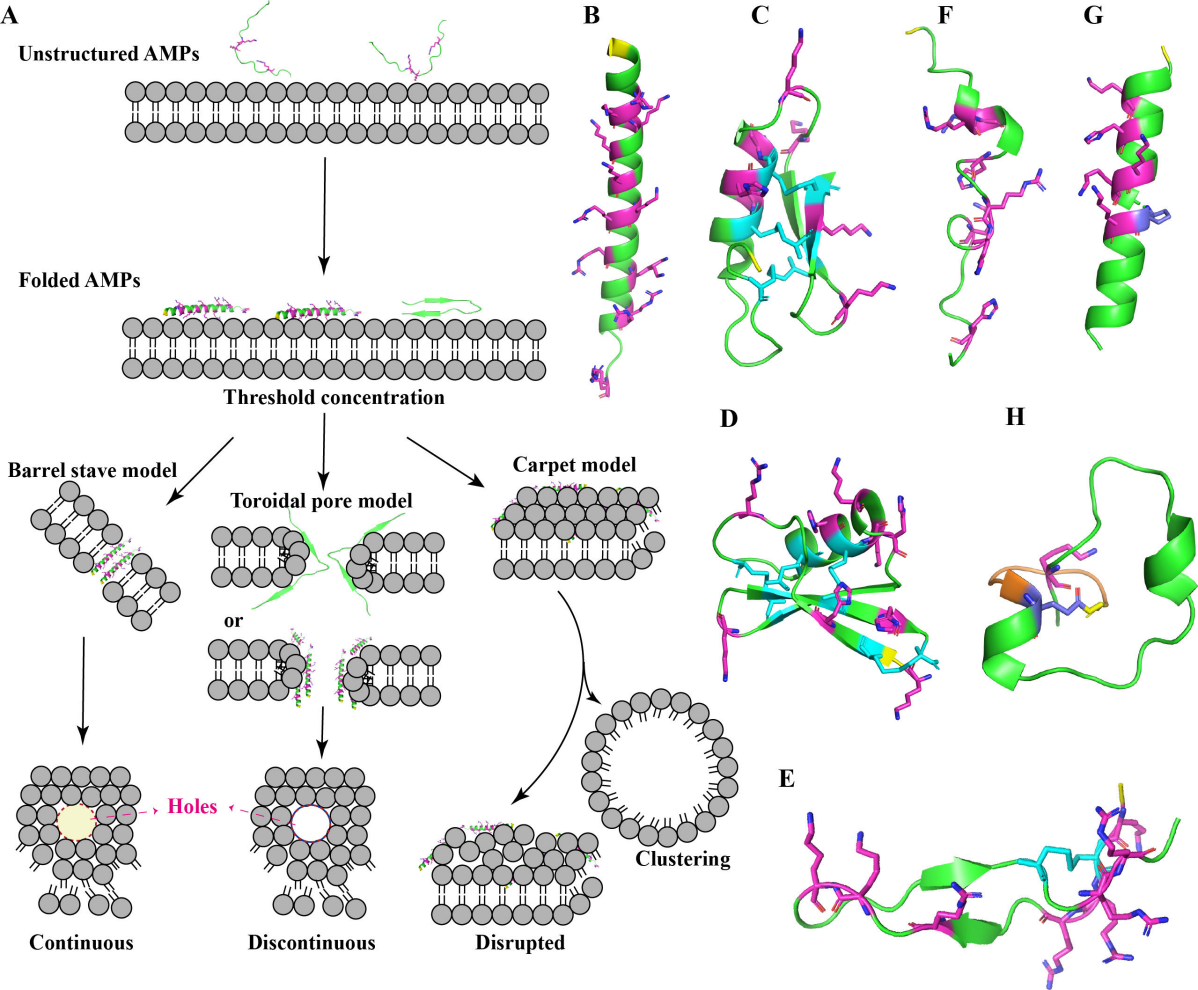
Cartoons illustrating the mechanism of action of membrane-targeting AMPs and the structures of several representative AMPs. (**A**) AMPs are typically unstructured in solution but undergo rapid folding to adopt the favorable conformation for interaction with the membrane bilayer. Subsequently, depending on their biophysical properties, the membranes can be destabilized through one of three mechanisms: the barrel-stave model (left), the toroidal pore model (middle), or the carpet model (right). Notably, the structures shown (e.g., β-sheet and α-helical peptides) are representative examples to illustrate the mechanisms and are not exclusive to these conformations. (**B–H**) Cartoons depict the conformations of (**B**) LL-37 (PDB ID: 5NMN), (**C**) plectasin (PDB ID: 3E7U), (**D**) plant defensin (AlphaFold ID: AF-B3EWP3-F1), (**E**) lactoferricin B (PDB ID: 1LFC), (**F**) pleurocidin (PDB ID: 6RSF), (**G**) magainin 2 (PDB ID: 5CGO) and (**H**) achromonodin-1 (PDB ID: 8SVB, residues in the ring are highlighted in orange). In these illustrations, positively charged residues (Arg, His, and Lys) and Glu are represented as pink and blue sticks, and disulfide bonds are highlighted in cyan. The initial residues (non-C-terminal) of these AMPs are indicated by the yellow sticks. The images were created using ChemDraw and PyMOL. PDB ID refers to the unique identifier assigned to a protein in the Protein Data Bank.

According to their targeted pathogens, AMPs are also often classified as antibacterial, antifungal, antiparasitic, and antiviral peptides and so on (8). Nowadays, this target specificity not only applies to therapeutic roles in treating infections in clinical settings but may also be relevant in other applications, like plant protectants in agriculture. An example is defensin PDF1.2, found in *Arabidopsis thaliana*, which has been introduced into various plants through transgenic technology to enhance their disease resistance (21). Beyond their functional classifications, AMPs can also be further characterized by their intrinsic physicochemical properties. In terms of the residues, many of the reported AMPs have a high percentage of residues with the positive charge (average net positive charge in AMPs: 3.32) (8), primarily due to the presence of basic amino acids such as histidine (His, H), arginine (Arg, R), and lysine (Lys, K), although there are some neutral or even anionic AMPs such as daptomycin and chrombacin (8, 22). An extreme example is novexatin, a cyclic cationic peptide composed entirely of seven arginine residues (RRRRRRR) (23). This cationic property facilitates interactions with the negatively charged components of bacterial membranes. Meanwhile, many AMPs adopt either an α-helical (like magainin 2) (24) or β-sheet (like lactoferricin B) (25) conformation in membrane mimicking environments, although variations in their secondary structures can also occur (8) (Fig. 1A). This structural adaptability allows them to vertically embed in the cell membrane and subsequently bend to form a ring hole (α-helical) or align parallel to the cell membrane, with their hydrophobic ends facing the phospholipid bilayer (α-helical and/or β-sheet) (26) (Fig. 1A). Besides, it is often observed that these AMPs contain hydrophobic amino acids, such as tryptophan (Trp) and glycine (Gly) (8). This hydrophobic character is crucial for disrupting the integrity of microbial membranes, as it enables AMPs to attach to or insert themselves into the hydrophobic lipid bilayers of cell membranes. In this context, these AMPs are often also classified into several subgroups, like Trp-rich peptides (tritrpticin) (27), Gly-rich peptides (diptericin) (28). Indeed, the primary mechanism by which AMPs exert their antimicrobial effects is through the disruption of microbial membranes (8). In view of this, one of the most promising advantages of AMPs is their relatively low toxicity to mammalian cells (29), which stems from the differences in membrane composition between host cells and bacteria, including pathogens. While bacterial membranes are rich in negatively charged phospholipids, mammalian membranes contain more neutral lipids, enabling AMPs to preferentially target bacteria (30).

Moreover, the cell wall is another target for AMPs (31). Certain AMPs, such as lantibiotics, specifically target enzymes involved in bacterial cell wall synthesis, including transpeptidase, to inhibit the cross-linking of peptidoglycan, ultimately resulting in cell death (32). Another example is friulimicin B, a naturally occurring AMP produced by *Actinoplanes friuliensis*. This AMP, in the presence of calcium, forms the unique complex with a lipid carrier that is not targeted by other antibiotics currently in use, which prevents the formation of a functional cell wall, leading to strong cidal activity against gram-positive pathogens (33). Furthermore, recent research has indicated that some AMPs may interfere with bacterial signal transduction pathways that regulate cell wall synthesis. For example, a cyclic di-GMP sequestering peptide (CSP) can influence secondary messenger systems, such as cyclic di-GMP (34), in bacteria, thereby disrupting their growth and biofilm formation (35). Besides the aforementioned processes, other studies have reported that targeted processes include, but are not limited to, cell division (MciZ), protein synthesis (proline-rich AMP Tur1A), nucleic acid degradation (TFP1-1TC24), and protease activity (cathelicidin-BF) (8, 36–38).

Beyond these direct antimicrobial activities, certain HDPs might further play a role in modulating the immune response (8). They can recruit immune cells to the site of infection and enhance the overall immune response. One notable example is LL-37, a human cathelicidin composed of 37 amino acids, which is usually detected in the skin of newborn infants (Fig. 1B; Table 1) (39). It attracts various immune cells, such as monocytes and neutrophils, to the site of infection, thereby enhancing the local immune response (40). Furthermore, LL-37 has been observed to stimulate the activity of macrophages and dendritic cells, promoting the production of cytokines, which facilitates the adaptive immune response and improves the body’s ability to resist infections (41). Additionally, LL-37 can regulate inflammatory responses, helping to control excessive inflammation and protect tissues from damage (41).

**TABLE 1:** Sequence analysis of several representative AMPs exemplified in the review (excluding complex peptides such as lipopeptides and glycopeptides)^*a*^

AMP	Sequence	Percentage of (R + K + H)	pI	GRAVY value	No. of cysteines	Target	Reference
Tryglysin A	VNSWGKH	28.57	9.70	−1.214	0	G^+^	(42)
BP100	KKLFKKILKYL-NH_2_	45.45	11.02	−0.191	0	G^−^	(43)
Cationic peptide A	KPQQHNRPLRHK	50.00	12.53	−2.758	0	G^+^, G^−^	(44)
Tritrpticin	VRRFPWWWPFLRR	30.77	12.98	−0.792	0	Fungus, parasite, cancer cell, G^+^, G^−^	(45)
PGLa	GMASKAGAIAGKIAKVALKAL-NH_2_	19.05	11.28	0.843	0	Fungus, parasite, G^+^, G^−^	(46)
Pexiganan	GIGKFLKKAKKFGKAFVKILKK	40.91	11.70	−0.159	0	Cancer cell, fungus, G^+^, G^−^	(47)
Magainin 2	GIGKFLHSAKKFGKAFVGEIMNS	21.74	10.80	0.083	0	Fungus, virus, cancer cell, parasite, G^+^, G^−^	(48, 49)
P113	DSHAKRHHGYKRKFHEKHHSHRGY	58.33	10.89	−2.454	0	Fungus, virus, G^+^, G^−^	(50)
Pleurocidin	GWGSFFKKAAHVGKHVGKAALTHYL	28.00	10.87	−0.068	0	Cancer cell, fungus, G^+^, G^−^	(51)
Melittin	GIGAVLKVLTTGLPALISWIKRKRQQ	19.23	12.55	0.273	0	Fungus, virus, cancer cell, parasite, G^+^, G^−^	(52)
Cathelicidin-BF	KFFRKLKKSVKKRAKEFFKKPRVIGVSIPF	40.00	12.35	−0.537	0	Cancer cell, fungus, G^+^, G^−^	(53)
Achromonodin-1	GGGGPTPEYFLMPIDPAWLQANLPNTGKYN	3.33	4.18	−0.520	0	G^−^	(54)
NEMURI	DARARRIVRAGRRRGGRRGGRRGGRRSARKS	48.39	13.28	−1.900	0	G^−^	(55)
LL-37	LLGDFFRKSKEKIGKEFKRIVQRIKDFLRNLVPRTES	19.57	11.35	−0.724	0	Fungus, virus, cancer cell, parasite, G^+^, G^−^	(56)
Cecropin A	KWKLFKKIEKVGQNIRDGIIKAGPAVAVVGQATQIAK	21.26	11.18	−0.073	0	Fungus, virus, cancer cell, parasite, G^+^, G^−^	(57)
*Drosophila* Diptericin	DDMTMKPTPPPQYPLNLQGGGGGGSGDGFGFAV QGHQKVWTSDNGRHEIGLNGGYGQHLGGPYGNSEPSWKVGSTYTYRFPNF	9.64	6.80	−0.884	0	G^+^	(58)
Attacin	LPQNNIADDDFQEVQRYSSRIIDPGSQFLIRGEDL DDIFEPREEEGLPEDVIRARRSPQDGRRGSASVT VNNESRRGTDVRADLNARLWEGNNRRSSLDANAYYQRHFG	15.60	4.63	−1.137	0	G^−^	(59)
hLF1-11	GRRRRSVQWCA	36.36	12.50	−1.373	1	G^+^, G^−^	(60)
Ranacyclin E	SAPRGC^1^WTKSYPPKPC^1^K	23.53	10.14	−1.218	2	Fungus, G^+^, G^−^	(61)
Ranacyclin T	GALRGC^1^WTKSYPPKPC^1^K	23.53	10.14	−0.876	2	Fungus, G^+^, G^−^	(61)
Lactoferricin B	FKC^1^RRWQWRMKKLGAPSITC^1^VRRAF	32.00	12.35	−0.576	2	Fungus, virus, cancer cell, G^+^, G^−^	(62)
Nv-CATH	NC^1^NFLC^1^KVKQRLRSVSSTSHIGMAIPRPRG	23.33	11.56	−0.363	2	G^+^, G^−^	(63)
Sakacin *P*	KYYGNGVHCGKHSCTVDWGTAIGNIGNNAAA NWATGGNAGWNK	11.63	8.77	−0.574	2	G^+^	(20)
Enterocin O16	LGSCVANKIKDEFFAMISISAIVKAAQKKAWKEL AVTVLRFAKANGLKTNAIIVAGQLALWAVQCGLS	13.24	10.58	0.622	2	Fungus, G^+^	(64)
Iseganan	RGGLC^1^YC^2^RGRFC^2^VC^1^VGR	23.53	9.08	0.241	4	G^+^, G^−^	(65)
Protegrin-1	RGGRLC^1^YC^2^RRRFC^2^VC^1^VGR	33.33	10.92	−0.250	4	Fungus, virus, G^+^	(66)
Plectasin	GFGC^1^NGPWDEDDMQC^2^HNHC^3^KSIKGYKGGYC^1^AKGGFVC^2^KC^3^Y	17.50	7.78	−0.695	6	Fungus, virus, G^+^	(67)
Human β-defensin 2	GIGDPVTC^1^LKSGAIC^2^HPVFC^3^PRRYKQIGTC^2^GLPGTKC^1^C^3^KKP	19.51	9.19	−0.102	6	Fungus, virus, G^+^, G^−^	(68)
Human β-defensin 3	GIINTLQKYYC^1^RVRGGRC^2^AVLSC^3^LPKEEQIGKC^2^STRGRKC^1^C^3^RRKK	28.89	10.63	−0.700	6	Fungus, virus, cancer cell, G^+^, G^−^	(68)
Hepcidin	DTHFPIC^1^IFC^2^C^3^GC^4^C^1^HRSKC^2^GMC^3^C^4^KT	20.00	7.98	0.388	8	Fungus, G^+^, G^−^	(69)

^*a*^ The cysteines involved in the same disulfide bond are highlighted in the same superscripts. The net charge and hydrophobicity of the sequences are indicated by their isoelectric points (pI) and protein GRAVY values, respectively, where higher GRAVY values exhibit a positive correlation with increased hydrophobicity. G^+^ denotes gram-positive bacterium, while G^−^ refers to gram-negative bacterium.

Notably, the interaction between AMPs and their targets is very complex, involving various regulatory mechanisms employed by both bacteria and host cells. Bacteria have evolved numerous strategies to evade the effects of AMPs. One common mechanism is the alteration of their cell membrane composition. For instance, gram-negative bacteria can modify their lipopolysaccharides to reduce the binding affinity of cationic AMPs. A typical example is *Escherichia coli*, which expresses mobile colistin resistance enzymes that enable these bacteria to resist cationic AMPs such as colistin (1). This expression can lead to a less negatively charged surface, which repels positively charged AMPs, thereby diminishing their effectiveness. Similarly, *Staphylococcus aureus* can modify its membrane lipid composition by altering the proportion of anionic lipids such as cardiolipin, leading to resistance against the short snake-derived antimicrobial peptide ATRA-1 (70). Another strategy employed by bacteria is the production of enzymes that degrade AMPs. For example, certain strains of *Pseudomonas aeruginosa* produce proteases, such as alkaline protease A and elastase B, which are capable of cleaving AMPs, rendering them inactive (71). This enzymatic degradation presents a significant challenge in treating infections caused by resistant bacteria. Additionally, some bacteria can secrete AMP-binding proteins that sequester AMPs, preventing them from interacting with their target sites. A typical phenomenon has been observed in multidrug-resistant *Acinetobacter baumannii*, which produces AbOmpA that binds to and neutralizes the activity of LL-37 against this pathogen (72). Besides, some bacteria including *S. aureus* can express *vraFG* transporter, which functions to transport AMP in the *aps* AMP sensor/regulator system (73). Notably, bacteria can also communicate through quorum sensing, secreting signaling molecules (e.g., auto-inducing peptides in *Cryptococcus neoformans*) to coordinate behaviors like biofilm formation and virulence factor production (74, 75). Biofilms create a protective barrier, reducing AMP penetration and enhancing bacterial survival (74). At critical densities, bacteria can also upregulate AMP resistance genes via quorum sensing or quenching. For instance, enterocin O16, an AMP from *Enterococcus faecalis*, is regulated by the fsr quorum-sensing system (76).

Meanwhile, host cells actively regulate AMP expression and activity during infection. Immune cells like macrophages and neutrophils upregulate AMPs such as cathelicidin (e.g., LL-37) and defensins (e.g., β-defensins) upon pathogen encounter (77, 78). Beyond immune cells, epithelial cells also contribute significantly. For example, skin keratinocytes produce β-defensin 3 to enhance barrier function (79), while respiratory epithelial cells release human β-defensin 2 and 3 during infections (80, 81). These interactions highlight the complexity of host-pathogen dynamics and the challenges in developing effective antimicrobial therapies, emphasizing the need to understand these mechanisms to combat AMR.

## ADVANTAGES AND DISADVANTAGES OF AMPs, AND EMERGING STRATEGIES FOR FUTURE APPLICATIONS OF AMPs

Compared to conventional antibiotics that refer to widely used small-molecule antimicrobial agents without typical peptide linkages in clinical practice and molecular weight that is typically less than 500 Dalton, such as β-lactams, tetracyclines, and quinolones, AMPs represent a distinct class of antimicrobial agents. As mentioned earlier, AMPs can be divided into the following subsets based on their source: bacteriocins derived from bacteria and HDPs produced by a wide range of host cells (16). Bacteriocins typically exhibit a narrow spectrum of activity, which enhances their specificity in clinical applications. Interestingly, HDPs typically exhibit broad-spectrum activity against a wide range of pathogens, including bacteria, fungi, and even some viruses. This broad-spectrum efficacy is particularly valuable in treating polymicrobial infections, where multiple pathogens are involved. A notable example is LL-37, which has been reported to be effective in treating multimorbidity, including keratitis (82), psoriasis (82), and severe acute respiratory syndrome coronavirus 2 (SARS-CoV-2) infection (83). Besides, these HDPs possess additional immunomodulatory properties. Conventional antibiotics, in contrast, do not typically have immunomodulatory effects. It is worth noting that some conventional antibiotics might also be peptides, such as polymyxins and vancomycin. However, unlike HDPs which exhibit diverse mechanisms of action, these peptides primarily target specific bacterial components, such as the cell wall or membrane. Thus, compared to conventional antibiotics, which often target single bacterial pathways and face rapid resistance development (1, 84), AMPs might exhibit a multimodal mechanism of action. This makes it significantly more challenging or slower for bacteria to evolve resistance, a crucial advantage in the context of rising AMR. This makes them advantageous candidates for combination therapies involving AMPs as well as for their monotherapy (85). Indeed, recent studies have revealed that various conventional antibiotics can be re-sensitized to their resistant pathogens by synergizing with certain AMPs (85). A notable example is last-resort antibiotic colistin, whose efficacy is compromised by the global spread of mobile colistin resistance genes (86, 87). To address the issue, Cirioni’s group and Douglas’s group independently discovered that colistin activity can be restored by combining it with LL-37 (88) and CDP-B11 (89), respectively.

Besides, some AMPs typically exhibit a rapid mechanism of action by dominantly disrupting bacterial cell membranes within minutes of exposure. An example is GATR-3, which completely killed *Acinetobacter baumannii* within 1 hour (the time can be shortened into 10 minutes if 5- and 10-fold minimal inhibitory concentrations of GATR-3 are used) (90). Such swift action can be crucial in clinical settings, particularly in cases of severe infections where time is of the essence. In contrast, conventional antibiotics often require longer periods to exert their effects, allowing pathogens to proliferate and potentially exacerbate the infection.

Notably, many AMPs are derived from natural sources, such as the human gut microbiome. This natural origin can be advantageous in terms of safety and biocompatibility, as these peptides are often less toxic to human cells compared to synthetic antibiotics. Six months ago, Santos and his colleagues analyzed the global microbiome using machine learning, identifying 63 peptides that exhibited targeted activities against pathogens while maintaining high safety profiles (91).

However, despite these advantages, there are numerous challenges that AMPs face before they can be practically applied. One of the primary challenges associated with AMPs is their stability. Many AMPs are susceptible to degradation by proteolytic enzymes in biological environments, which can limit their effectiveness *in vivo*. A notable example is P113, a histatin-derived AMP with antifungal activity, which is susceptible to protease degradation in human saliva (92). In contrast, conventional antibiotics, particularly those that are chemically synthesized, often exhibit greater stability and longer half-lives, allowing for sustained therapeutic effects. Meanwhile, delivering AMPs to the site of infection can be challenging due to their size and charge. Many AMPs are cationic and may interact with negatively charged biological membranes, which can influence their distribution and bioavailability (8). An example can be found in human β-defensin 3, where its charge significantly affects its bioavailability (93). In contrast, conventional antibiotics, particularly those formulated for specific delivery routes, often have more straightforward administration protocols. Moreover, due to AMP’s relatively high molecular weights and significant polar surface area, their diffusion into the bloodstream might be further impeded (8). Thus, to date, nearly all approved AMPs for sale in the market have been administered parenterally, particularly via intravenous routes, instead of oral administration.

Transdermal delivery, a non-invasive method leveraging the skin’s accessibility and barrier properties, is an increasingly prominent alternative to mitigate systemic side effects and ensure sustained release (94). Biomaterial-based systems, such as nanofibers, hydrogels, liposomes, and nanoparticles, have become essential for enhancing transdermal AMP delivery efficacy (95, 96). A recent representative example involves five amphiphilic α-helical AMPs (MAP, BP100, TP10, PGLa, and MSI-103) that were site-selectively conjugated to gold nanoparticles via a cysteine-pentaglycine linker, significantly improving proteolytic stabilities while maintaining biological activities in their delivery processes (97). Meanwhile, peptides themselves represent another valuable area for improvement (97). Specifically, chemical modifications, such as cyclization, pegylation, incorporation of non-natural amino acids, and lipidation, can enhance the stability and antimicrobial activity of AMPs (95, 96). One notable example is cyclic peptides (98), which have demonstrated increased resistance to proteolysis and enhanced membrane-disrupting capabilities. Meanwhile, considering that many proteinases exhibit high activity against L-amino acids, particularly residues such as Asp (aspartate), Asn (asparagine), and Met (methionine), methylation and acetylation are often employed to modify the N-terminal. In special cases, these modifications may involve replacing L-amino acids with D-amino acids or residue mutation (99). Notably, although various chemical modifications can be performed to improve stability, it remains uncertain whether these alterations will affect their biological activity. This area may become a significant focus for future research. Meanwhile, computational modeling and machine learning are extensively utilized to rapidly screen and refine potential candidates prior to peptide synthesis (100). Two months ago, Zhao and her co-workers reported a model called deepAMPNet, which utilizes structures of AMPs predicted by AlphaFold2 to encode residue-level features through a bidirectional long short-term memory protein language model (101). This technology constructs adjacency matrices based on the contact maps of amino acids and facilitates the refinement of AMPs.

Additionally, the production of AMPs might be more complex and costly compared to conventional antibiotics. Besides, it typically requires advanced techniques, such as recombinant DNA technology or solid-phase peptide synthesis (SPPS), which may further increase production challenges. These factors may limit the scalability and affordability of AMPs for widespread clinical use (102). Indeed, the cost per gram of peptide varies depending on various factors, such as the synthesis methods employed. It is estimated that the production costs of host defense peptides are nearby €250,000 per gram when using recombinant expression systems, while the costs for those produced through chemical synthesis may be even higher (103, 104). Researchers are investigating alternative production methods, such as recombinant DNA technology and microbial fermentation, to lower costs and enhance yield. Rose et al. developed a novel, scalable auto-induction strategy utilizing a specially designed culture medium to significantly boost peptide yields of GKY20 and ApoB, and reduce production costs to €42,000 per gram (103).

Another potential concern regarding AMPs is their potential cytotoxic effects at higher concentrations, despite many being derived from natural sources and generally considered safe. A notable example is melittin, the main active component of honeybee (*Apis mellifera*) venom. This AMP exhibited relatively low cytotoxicity in various cell lines when administered at the minimal inhibitory concentration of 1.44 ± 0.11 µM against *S. aureus*. However, higher concentrations would inhibit the growth of HEK293 cells, as its half-maximal inhibitory concentration for HEK293 cells was 9.67 ± 3.63 µM (105). This potential for toxicity raises significant concerns about their applied dosages in clinical settings, particularly for patients with compromised immune systems or underlying health conditions. While traditional antibiotics are not without side effects, they often possess well-established safety profiles. Future research should focus on understanding how to mitigate the side effects associated with this class of AMPs. Ongoing research aims to identify and develop AMPs with a favorable therapeutic index, minimizing harm to healthy normal flora while effectively targeting pathogens. A typical example is the competence-stimulating peptide, which serves as the targeting domain for selectively targeted AMPs. This AMP can effectively eliminate *Streptococcus mutans* from multispecies biofilms without adversely affecting closely related non-cariogenic oral streptococci, thereby preserving the protective benefits of a healthy oral flora for future clinical applications (106).

Moreover, despite the considerable interest in AMPs, the number of clinical trials and approved AMP-based therapies remains relatively limited when compared to traditional antibiotics (107). This scarcity of extensive clinical data can impede the acceptance and adoption of AMPs in clinical practice, as healthcare providers may be more accustomed to established antibiotic therapies. Indeed, the regulatory pathway for approving antimicrobial peptides may be also complex and lengthy.

Nowadays, the therapeutic potential of AMPs has been investigated across various fields, including wound healing, oral health, and the treatment of systemic infections (8). One of the most promising applications of AMPs is in addressing antibiotic-resistant infections (29). Due to their unique characteristics, i.e., multiple mechanisms of action, rapid efficacy, excellent bactericidal activity, and lower selective pressure (108), AMPs present a novel strategy for combating drug-resistant pathogens, and several AMPs have demonstrated effectiveness against methicillin-resistant *Staphylococcus aureus* (MRSA) and vancomycin-resistant *Enterococcus* (VRE) (109). A notable example is LL-37, which successfully re-sensitizes colistin to colistin-resistant strains (88). Additionally, the immunomodulatory properties of AMPs make them attractive candidates for combination therapies, where they can be used alongside traditional antibiotics to enhance efficacy and mitigate the development of resistance, particularly in immunocompromised patients (110). An example is protegrin-1 (PG-1) derived from porcine leukocytes, which exhibits a synergistic interaction with colistin against *Acinetobacter baumannii*. Given the high incidence of hospital-acquired infections caused by *Acinetobacter baumannii*, particularly in immunocompromised patients, PG-1 has been identified as a promising antimicrobial agent for these cases (111). Meanwhile, the application of AMPs in the development of novel antimicrobial coatings for medical devices and implants is also gaining traction (112). These coatings can provide a sustained release of AMPs, preventing biofilm formation and reducing the risk of device-related infections (113). Moreover, the integration of AMPs into personalized medicine is an emerging trend (112). For example, mutations in genes, including those encoding hepcidin AMP, are being integrated to facilitate early genetic screening, diagnosis, and personalized management strategies for patients with hereditary hemochromatosis (114). Notably, the emergence of resistance to AMPs, while less prevalent than with conventional antibiotics, remains a significant concern.

## CURRENT LANDSCAPE OF AMP APPLICATIONS, RECENTLY IDENTIFIED REPRESENTATIVES, AND MARKET-APPROVED TRENDS

AMPs have significant applications across various fields, including medicine, agriculture, and food safety. However, before their use, these numerous AMPs must undergo clinical trials (Table 2) (8). One current example is the linear tetrapeptide EA-230, which is derived from human chorionic gonadotropin and is in phase I and II clinical trials (22, 115). Meanwhile, some AMPs have already received approval from the relevant regulatory authorities, such as the USA (FDA: Food and Drug Administration) and the European Union (EMA: European Medicines Agency), for specific applications, such as wound healing for LL-37 and topical antiseptics for pexiganan cream (17, 116). Beyond their antibiotic applications, AMPs are also being explored for their potential in treating various diseases including cancer and inflammatory diseases, and even as adjuvants in vaccine formulations against diseases like tuberculosis (8, 117). For instance, iseganan, a protegrin-1 analog AMP, has demonstrated safety and potential efficacy in reducing ulcerative oral mucositis and its associated symptoms in clinical trials (118). Furthermore, AMPs can also serve as natural pesticides, such as attacin (an AMP isolated from silkworm), to protect crops from bacterial and fungal infections, thereby reducing reliance on synthetic pesticides and promoting sustainable agricultural practices (119). In livestock, AMPs such as cathelicidin can be employed to prevent infections and enhance growth, which diminishes the need for antibiotics in animal husbandry (120). Additionally, incorporating AMPs into food packaging materials can provide an extra layer of protection against microbial contamination (121). For example, a study demonstrated that food packaging films composed of poly-(epsilon-caprolactone) blended with nisin could effectively inhibit the growth of *S. aureus* and *E. coli* on fruit surfaces. These antimicrobial fibrous mats extended the fruits’ freshness by reducing microbial colonization, thereby significantly improving shelf life under refrigerated conditions (122). Currently, researchers are investigating ways to mimic the structure and function of AMPs to design new materials with antimicrobial properties (121). These studies of AMPs have inspired the development of biomimetic materials and applications in nanotechnology.

**TABLE 2:** Some representative AMPs in various clinical phases^*a*^

AMP	Indications	Status	Phase or type	ClinicalTrials.gov ID
Daptomycin	MRSA bloodstream infection; MRSA bacteremia; cellulitis; wound infections	Recruiting; completed; completed; completed	Phase IV; phase III; phase IV; phase IV	NCT06637332; NCT01898338; NCT00295178; NCT01080963
Defensin	Skin aging	Completed	Phase IV	NCT02765763
EA-230	Systemic inflammatory response syndrome; endotoxemia and systemic inflammatory response	Not yet recruiting; completed	Phase II; phase I	NCT03145220; NCT02629874
Enfuvirtide	HIV infections; HIV infections; HIV infections	Completed; completed; completed	Phase IV; phase III; phase II	NCT00187551; NCT00529243; NCT00333736
Friulimicin B	Acquired pneumonia and skin infections	Terminated	Phase I	NCT00492271
Gramicidin	Hordeolum	Recruiting	Phase III	NCT00534391
Hepcidin	Breast or gynecological cancer; Gaucher disease; chronic periodontitis; chronic kidney disease stage 1–2	Recruiting; recruiting; completed; completed	Observational; observational; phase IV; phases II/III	NCT06483997; NCT02437396; NCT02641210; NCT06706271
hLF1-11	Bacterial and fungal infections	Withdrawn	Phases I/II	NCT00430469
Iseganan	Pneumonia; head and neck cancer	Terminated; not yet recruiting	Phases II/III; phase III	NCT00118781, NCT00022373
Lactoferrin	Critical illness; HIV infections; antibiotic-associated diarrhea; sepsis	Not yet recruiting; completed; completed; recruiting	Phase IV; phase II; phase II; phases II/III	NCT05936528; NCT01830595; NCT02626104; NCT06181422
LL-37	Melanoma; *Shigellosis* infection; diabetic foot ulcer	Completed; completed; not yet recruiting	Phases I/II; phase II; phase II	NCT02225366; NCT00800930; NCT04098562
Magainin	Infected diabetic ulcers; diabetic foot ulcers	Completed; completed	Phase III; phase III	NCT00563433; NCT00563394
Nisin	Oral cavity squamous cell carcinoma	Recruiting	Phases I/II	NCT06097468
Novexatin	Onychomycosis	Completed	Phase II	NCT02933879
P113	Oral candidiasis	Completed	Phase II	NCT00659971
Peginesatide	Anemia and chronic kidney disease; anemia	Completed; terminated	Phase III; phase IV	NCT01478971; NCT01737879
Pexiganan	Diabetic foot infection; diabetic foot infection; diabetic foot ulcers; diabetic foot ulcers	Completed; completed; completed; completed	Phase III; phase III; phase III; phase III	NCT01594762; NCT01590758; NCT00563433; NCT00563394
Polymyxin B	Sepsis; CRAB infections; sepsis and septic shock	Completed; recruiting; completed	Phase III; phase III; phase IV	NCT00490477; NCT06440304; NCT00629382
Polymyxin E	Gram-negative infections or sepsis; gram-negative infections; nosocomial infections; ventilator-associated pneumonia	Completed; recruiting; recruiting; not yet recruiting	Phase IV; phase III; phases II/III; phase IV	NCT03397914; NCT05613361; NCT06650384; NCT05922124
PXL01	Surgical adhesions	Completed	Phase II	NCT01022242
Vancomycin	VRE colonization; *Clostridium difficile* infection; lumbar fusion surgery	Recruiting; completed; not yet recruiting	Phases I/II; phase IV; phases II/III	NCT05715619; NCT02951702; NCT06748144

^*a*^ All details were collected from https://clinicaltrials.gov/ (accessed on 24 February 2025). All AMPs exemplified in the review are checked, and only AMPs with available data on the website are included in the table. For those AMPs in clinical trials, several representative studies are selected and listed.

In addition to the aforementioned AMPs, scientists have utilized a variety of techniques throughout the extensive history of AMP research to uncover a series of widely used AMPs. Nisin A, one of the first recognized AMPs, was discovered in the 1920s from *Lactococcus lactis* by Rogers and Whittier (8). This 34-amino-acid peptide contains unusual residues like lanthionine and methyllanthionine (123), forming loops and helical regions that interact with lipid II, disrupting the cell wall of gram-positive bacteria. Nisin A also forms pores in bacterial membranes, causing ATP and ion leakage, enhancing its bactericidal activity (124). Nisin A and its variants, like nisin Z, are widely used as food preservatives and are FDA-approved as Generally Recognized as Safe substances (125). Plectasin, derived from the fungus *Pseudoplectania nigrella*, is a 40-amino-acid peptide that strongly interacts with lipid II, forming a dense, Velcro-like structure on bacterial membranes (Fig. 1C; Table 1) (31). It is a promising candidate for novel antibiotics, especially in addressing AMR. Defensins, another well-known AMP family, are categorized into subgroups, such as α-, β-, θ-, and γ-defensins based on structure, size, and disulfide bond patterns (8). They disrupt microbial membranes, inhibit cell wall synthesis, and interfere with microbial metabolism (126). Plant defensins, first identified in wheat and barley, are small cationic peptides with conserved cysteine motifs that stabilize their β-sheet structures (Fig. 1D; Table 1) (13, 127). These peptides play a key role in plant immunity and are being explored for agricultural and therapeutic applications, including vaccine development (8). Animal-derived defensins, such as human β-defensin 2, exhibit stage-specific expression and protect against microbial infections (39). Lactoferricin B, a bovine-derived peptide, is rich in basic residues and exhibits broad-spectrum activity against bacteria, fungi, and viruses (Fig. 1E; Table 1) (128). It disrupts microbial membranes and interferes with nucleic acid synthesis (128). Currently, it is investigated for immune enhancement and as a food preservative (129). PXL01 and hLF1-11, two synthetic peptides derived from human lactoferrin, show promising antimicrobial and antifungal activities, with PXL01 used to prevent post-surgical adhesions (130), while hLF1-11 has demonstrated a synergistic antifungal effect when combined with the conventional antibiotic fluconazole against *Malassezia furfur* CBS7019 (131). Pleurocidin, a 25-amino-acid peptide from winter flounder skin, has broad-spectrum activity against bacteria and fungi (Fig. 1F; Table 1) (132). Its amphipathic structure allows it to disrupt microbial membranes, making it a potential therapeutic for multidrug-resistant infections and wound healing (132). Magainin 2, discovered in the African toad (*Xenopus laevis*), is a 23-amino-acid peptide with an α-helical structure in membranes (Fig. 1G; Table 1) (8). Its amphipathic nature enables pore formation, leading to cell death (8). Magainin 2 and its analogs are being explored for wound care and as topical antimicrobial agents, particularly against antibiotic-resistant pathogens. These classic AMPs demonstrate a variety of mechanisms of action and structural features, making them valuable in real-world applications.

While classic AMPs have laid the foundation for antimicrobial therapy, the escalating AMR crisis has driven researchers to explore novel AMPs with enhanced efficacy and reduced limitations. Recent advancements in AMP discovery have unveiled a new generation of peptides, ranging from unique AMPs (such as those with unique structures and first-in-family discoveries) to multifunctional AMPs (such as those with anti-biofilm properties and roles in cross-disciplinary applications), and even engineered AMPs (such as those with artificial modifications like fusion of fragments), expanding their application potential.

In 2019, scientists discovered an Arg-rich cationic AMP with a net charge of 14 positive charges, named as NEMURI, in the brains of *Drosophila* that were suffering from bacterial infections (Table 1) (55). This AMP disrupts negatively charged cell membranes, leading to bactericidal activity against gram-negative bacteria such as *Serratia marcescens* and *E. coli*. Interestingly, it appears to provide a link between sleep and immune function, as it can be secreted ectopically to induce prolonged sleep and promote survival following infection. Nowadays, it has been widely used to explore the mechanisms behind increased sleep following infection, providing a reference for future clinical applications.

Cacaoidin, a serine-rich natural glycosylated lantibiotic AMPs, was identified from *Streptomyces cacaoi* as the first member of the new lanthidin RiPP family in 2020 (133). It contains approximately 40% hydrophobic residues out of a total of 23 residues, which facilitates the disruption of the cell membrane by binding to lipid II against gram-positive bacteria. Intramolecular thioether bonds and N-terminal modifications, including dehydration and glycosylation, were observed to enhance its biostability.

Besides, many AMPs with unique structures are also recently identified. Tryglysin A, a natural cyclic AMP identified from an oral *Streptococcus* strain isolated from wild rats in 2021, demonstrated excellent bactericidal activity against gram-positive bacteria (Table 1) (42). This peptide consists of seven amino acid residues, approximately 30% of which are hydrophobic. Notably, the side chains of α and δ carbons of Lys^6^ are fused with the six-membered ring of Trp^4^, forming an additional six-membered aliphatic ring. This unique structural modification results in an unprecedented macrocyclic configuration. Similarly, a natural lasso cyclic AMP, named achromonodin-1, was first isolated from *Achromobacter* in the sputum of patients with cystic fibrosis in 2024. It contains approximately 30% hydrophobic residues out of a total of 30 residues and forms an eight-membered macrolactam ring between Gly^1^ and Glu^8^ (glutamate^8^) via an isopeptide bond. The ring is further crossed by the remaining peptide chain, resulting in a unique structure that categorizes lasso peptides into three components, i.e., ring, tail, and loop (Fig. 1H; Table 1). Notably, this AMP has the largest experimentally determined loop structure consisting of 20 amino acids (54). The specific lasso structure confers achromonodin-1 with high stability and is responsible for its biological property as antimicrobial or other activity.

With advancing research, AMPs with multifunctional properties have been increasingly reported. Nv-CATH is a natural AMP isolated from the skin of frogs in 2022 (63). It consists of 30 amino acid residues and exhibits a net positive charge of 7, demonstrating 54.84% sequence similarity with frog cathelicidin-PY (Table 1). This AMP exhibits bactericidal activity against both gram-positive and gram-negative bacteria. Additionally, it possesses anti-inflammatory properties, as evidenced by its dose-dependent suppression of the gene expression of nitric oxide, interleukin-6, tumor necrosis factor-alpha, and interleukin-1 beta induced by lipopolysaccharide or lipoteichoic acid. Currently, it has been confirmed that Nv-CATH can significantly protect mice from lethal infections caused by intraperitoneally injected *S. aureus* (63). In the same year, Wang et al. identified a novel AMP named PEW300 and investigated its anti-biofilm properties against *Pseudomonas aeruginosa* (134). PEW300 exhibited strong antibacterial and anti-biofilm activity by preferentially dispersing mature biofilms, which led to the exposure and subsequent death of biofilm-encapsulated bacteria. Additionally, PEW300 demonstrated multiple mechanisms of action, including disrupting cell membrane integrity, inducing high levels of intracellular reactive oxygen species, and significantly reducing bacterial virulence.

Notably, scientists are increasingly interested in how to engineer AMPs by incorporating different domains. An example reported in 2025 is the novel multifunctional agent (8DSS-C8-P113), which consists of three domains: a non-specific AMP (P-113), a competence-stimulating peptide (C8), and an enhancing remineralization domain (8DSS). This engineered modification integrates the functional characteristics of individual AMPs and exhibits the ability to self-adhere to tooth surfaces without disrupting the normal oral flora. It also resists acid attacks, eradicates the biofilm of *Streptococcus mutans*, and induces mineralization to facilitate the repair of demineralized dental hard tissue (135).

**Fig 2:**
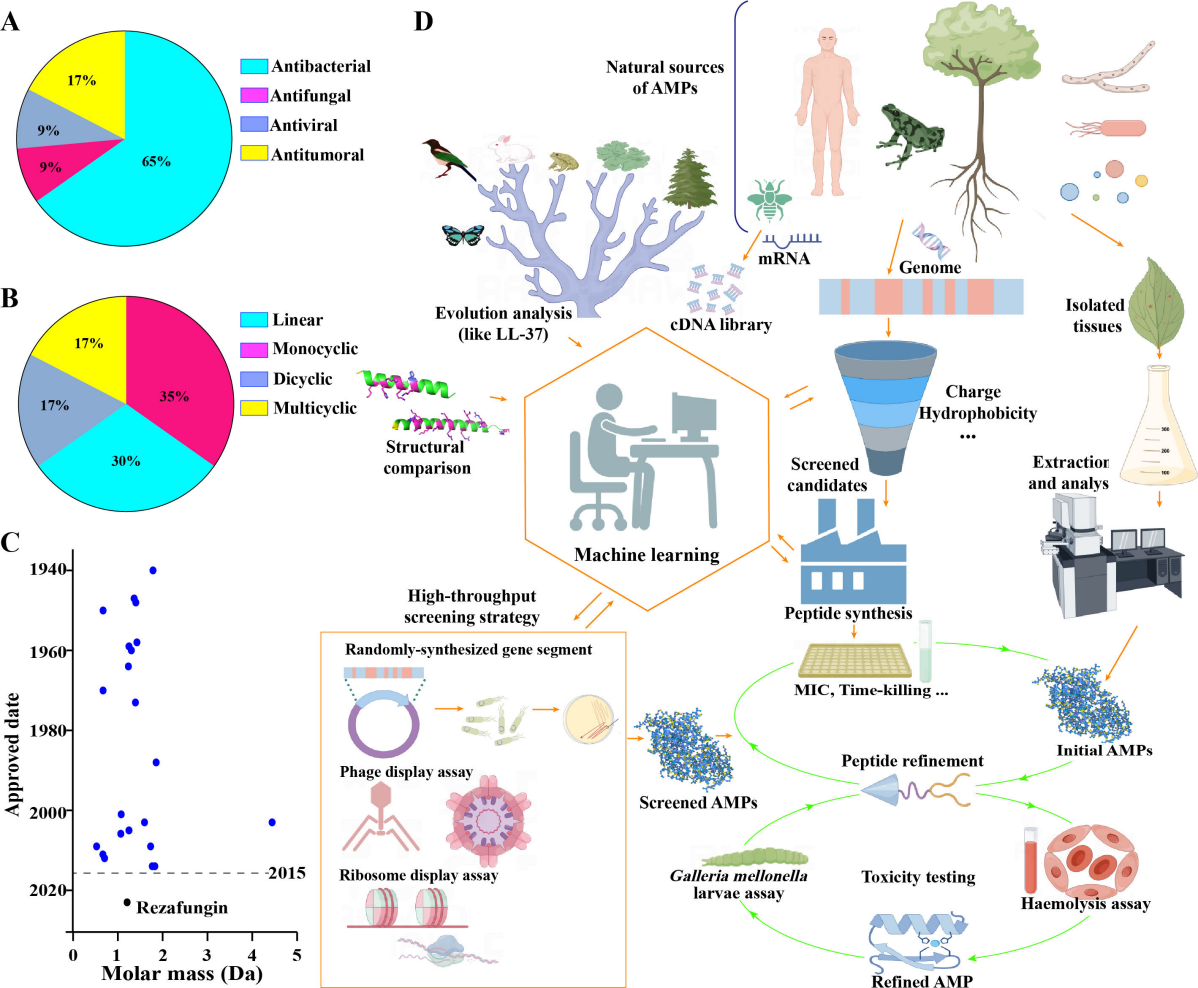
This analysis examines the approved AMPs and the general process for identifying potential AMPs. (**A, B**) The percentages of specific categories of these approved AMPs are classified according to (**A**) their functions and (**B**) the number of rings in their structures. (**C**) The distribution of molecular weights of these approved AMPs is illustrated. The only approved AMP within the last decade is rezafungin, which has been emphasized in black. (**D**) A flow diagram outlines the process for identifying AMPs. In addition to AMPs that are directly isolated from various sources, these peptides can also be predicted and identified through several direct methods, such as high-throughput screening strategies to collect peptides, or through indirect analyses using evolutionary and structural biological tools. Subsequently, further analysis of potential activity, toxicity levels, and additional refinement are conducted to isolate safe and effective AMPs. The image was created using Figdraw.

Since the 1980s, scientists have reported over 3,000 AMPs (136); however, over the past 25 years, only 12 AMPs have been approved by FDA or EMA for real-world therapeutic applications (137), with 11 of these approvals occurring a decade ago according to PepTherDia (http://peptherdia.herokuapp.com/), although there are additional AMPs that have been marketed in other countries but are yet to receive FDA or EMA approval. An example is enviomycin, which is marketed in Japan for the treatment of tuberculosis but lacks FDA or EMA approval (138). This observation aligns with a scientific principle, as it takes time to understand the underlying mechanisms before conducting clinical trials. Up to October 2024, fewer than 30 AMPs have been approved for sale in the market by the FDA and EMA (Fig. 2A through C; Table 3), with approximately 65% of them classified as antibacterial peptides (Fig. 2A). Meanwhile, the molecular weights of these AMPs primarily concentrate below 2 kDa (Fig. 2C). Another notable feature is cyclic peptides, which account for nearly 70% of these approved AMPs (Fig. 2B) (139). This trend is understandable because it is well-documented that cyclization can enhance the formation of intramolecular hydrogen bonds in peptides. This structural modification reduces solvation, increases the likelihood of membrane permeability, and provides greater resistance to proteolytic degradation (139). Additionally, cyclic peptides exhibit higher binding affinity to their targets due to reduced entropy loss. Thus, nearly all orally available peptides and highly bioactive natural products are predominantly cyclized.

**TABLE 3:** Approved AMPs available in the market, as sanctioned by the relevant regulatory authorities (FDA or EMA) since 2000^*a*^

Name of AMP		Sub-class	Main mechanism	Treated pathogen or cell type	Approved year	Reference
Brand	Generic					
Cancidas	Caspofungin	Lipopeptide (echinocandins)	Disrupt the cell wall by inhibiting β-(1,3)-D-glucan synthase	*Candida* and *Aspergillus* spp. and other fungi	2001	(140)
Cubicin	Daptomycin	Cyclic lipopeptide	Calcium-dependent membrane binding to disrupt cell membrane and/or cell wall	Gram-positive bacteria	2003	(141)
Fuzeon	Enfuvirtide	Linear peptide	Prevent viral fusion with host cell membranes by binding to HIV gp41 protein	Human immunodeficiency virus type 1	2003	(142)
Mycamine	Micafungin	Lipopeptide (echinocandins)	Disrupt the cell wall by inhibiting β-(1,3)-D-glucan synthase	Fungi (main *Candidiasis*)	2005	(143)
Ecalta	Anidulafungin	Lipopeptide (echinocandins)	Disrupt the cell wall by inhibiting β-(1,3)-D-glucan synthase	Fungi (main *Candidiasis*)	2006	(144)
Istodax	Romidepsin	Cyclic peptide	Kill cancer cells by inhibiting histone deacetylase	Cutaneous T-cell lymphoma	2009	(145)
Vibativ	Telavancin	Lipoglycopeptide	Disrupt the cell wall by binding to D-Ala-D-Ala	Gram-positive bacteria such as MRSA	2009	(146)
Incivek	Telaprevir	Linear peptide	Prevent viral polyprotein processing and replication by inhibiting HCV NS3/4A protease	Hepatitis C virus	2011	(147)
Kyprolis	Carfilzomib	Peptide epoxyketone	Inhibit the proteasome, leading to accumulation of misfolded proteins and apoptosis	Multiple myeloma cells	2012	(148)
Dalvance	Dalbavancin	Lipoglycopeptide	Disrupt the cell wall by binding to D-Ala-D-Ala	Gram-positive bacteria	2014	(149)
Kimyrsa	Oritavancin	Glycopeptide	Disrupt the cell wall and cell membrane	Gram-positive bacteria	2014	(150)
Rezzayo	Rezafungin	Lipopeptide (echinocandins)	Disrupt the cell wall by inhibiting β-(1,3)-D-glucan synthase	Fungi (*Candida* and *Aspergillus* spp.)	2023	(151)

^*a*^ These AMPs contain amino acids which are linked by typical peptide bonds (amide bonds). All data are collected from PepTherDia (http://peptherdia.herokuapp.com/), DrugBank (https://go.drugbank.com/), FDA (https://www.fda.gov), and EMA (https://www.ema.europa.eu) by searching for specific terms and filtering the results on a yearly basis. The cutoff date for these data is 23 October 2024.

Meanwhile, it is observed that a higher percentage of L-amino acids than D-amino acids exists in these approved AMPss. Interestingly, despite the presence of D-amino acids in short peptides of cell walls and specific antibiotics in the real world, they are less stable than L-amino acids. However, many proteinases exhibit high activity against L-amino acids in cellular environments. Consequently, the replacement of L-amino acids with D-amino acids can also be observed in specific cases of approved AMPs (99). Notably, to enhance the stability of AMPs, lots of modifications were observed at the terminals of these approved AMPs. Among them, nearly 60% exhibit modifications at their N-terminal, including acetylation, pyroglutamation, deamination, and cyclization. Meanwhile, their C-terminal is frequently amidated, cyclized, or subjected to other modifications (99). An example is WRK-30, a novel AMP derived from CLEC3A, which underwent truncation, glycine linker insertion, tryptophan addition, and D-amino acid modification to reduce cytotoxicity to eukaryotic cells, allowing for its use at higher concentrations in an *in vivo* setting (152).

It is worth noting that in the past 40 years, less than 50 AMPs have entered the clinical stage worldwide (99). Currently, more than 400 clinical trials involving AMPs such as nisin and daptomycin are in clinical use (https://clinicaltrials.gov/) (Table 2). Despite this, in some cases, certain clinical trials involving AMPs were discontinued or terminated for various reasons, with potential or unknown side effects being particularly noteworthy. The withdrawn cyclic peptide drug, Omontys (peginesatide), developed by Affymax and Takeda Pharmaceuticals, was recalled due to a fatal reaction that occurred after the first intravenous administration in nearly 0.02% of patients (approximately 25,000 individuals) (153). These situations also necessitate profound reflection.

## HOW TO COLLECT AND IDENTIFY AMPs

Understanding the pathway to discovering AMPs is crucial for their development. In earlier studies, researchers primarily collected AMPs from various organisms, including plants, animals, and microorganisms (154). They collected these samples to isolate and characterize AMPs using techniques such as fermentation and high-performance liquid chromatography (HPLC) before further evaluating their antimicrobial properties (Fig. 2D) (154). A typical example is ranacyclin E, which was directly isolated by Zhang et al. in 2021 from the skin secretion of the *Rana esculenta* frog (61). Mild electrical stimulation was used to collect the secretion, and then HPLC was further employed to separate them. This method to directly isolate AMPs from organisms offers natural diversity and proves bioactivity but is limited by low yields, complex extraction processes, and scalability challenges.

Nowadays, one of the most effective ways for identifying AMPs is through the use of bioinformatics tools and databases (155). Some of the most prominent databases include APD (Antimicrobial Peptide Database, https://aps.unmc.edu/AP/), dbAMP (Database of Antimicrobial Peptides, https://awi.cuhk.edu.cn/dbAMP/), DBAASP (Database of Antimicrobial Activity and Structure of Peptides, https://dbaasp.org/home), and DRAMP (Data Repository of Antimicrobial Peptides, http://dramp.cpu-bioinfor.org/) (8). Meanwhile, several computational tools can predict the antimicrobial potential of peptide sequences. These tools often use machine learning algorithms and various physicochemical properties to assess the likelihood of a peptide being an AMP. Specifically, machine learning and deep learning models are trained on known AMP data sets to predict novel AMPs from sequence databases. Among these tools, the AMP scanner (https://www.dveltri.com/ascan/) is a trained web-based method that predicts the antimicrobial activity of peptide sequences based on their physicochemical properties. Similarly, iAMP-2L (https://bio.tools/iamp-2l) and CAMP-R3 (http://www.camp3.bicnirrh.res.in/) are machine learning-based tools that predict AMPs using various physicochemical properties (8). Considering the significance of AMPs in maintaining a balance between their biostability in water-soluble environment and their hydrophobic characteristics that facilitate insertion into lipid bilayers, the proportion of hydrophobic residues is typically set at around 50% for most designed AMPs (129). Additionally, a high percentage of positively charged residues, such as Arg and Lys, is expected in proposed AMPs, as this cationic property enables interaction with the negatively charged components of bacterial membranes (129). Meanwhile, structural characteristics, such as the presence of anti-parallel β-sheet, are also expected to facilitate the ability of AMPs to interact with bacteria, which may also depend on cysteine-based disulfide bonds (156). Thus, structural biology techniques, including nuclear magnetic resonance (NMR), X-ray crystallography, and AlphaFold, are being integrated to elucidate and compare peptide structures for the development of antimicrobial motifs. By analyzing peptide sequences using these tools, researchers can quickly assess their potential as AMPs (Fig. 2D) (129). Song et al. applied these details to develop the iAMPCN model for predicting the activity of marine AMPs from the mucus of *Crassostrea gigas*, in which a total of 23 AMPs with significant antimicrobial potential were identified (157). In summary, by consulting these databases, researchers know potential AMPs by searching for specific sequences, structures, or properties.

Recently, evolutionary biology approaches are emerging as a powerful strategy for discovering novel AMPs by leveraging the evolutionary conservation of these peptides across diverse species. Many AMPs, such as defensins and cathelicidins, are highly conserved due to their critical role in innate immunity, making them ideal candidates for cross-species screening. For instance, the human cathelicidin LL-37 shares structural and functional similarities with its homologs in other vertebrates, highlighting the evolutionary conservation of AMPs. By analyzing homologous sequences and identifying conserved motifs, researchers can predict and validate potential AMPs in less-studied organisms. Last year, Wang et al. identified three novel cathelicidin AMPs from *Thamnophis sirtalis*, *Balaenoptera musculus*, and *Lipotes vexillifer*, which were subsequently designated as TS-CATH, BM-CATH, and LV-CATH, respectively (158). The advantage of this method lies in its ability to uncover functionally optimized AMPs that have been evolutionarily refined over time. Additionally, evolutionary conservation is often associated with low cytotoxicity and high stability (159), making these peptides promising candidates for therapeutic development.

Another significant point receiving increased attention is the studied trend of AMPs in lower animals, particularly insects, which rely heavily on AMPs for defense against microbes. They effectively accomplish this without the assistance of lymphocytes, a thymus, or antibodies (8). Thus, it is anticipated that these species harbor the unique and numerous collections of AMPs. The situation prompted scientists to re-examine these seemingly non-essential gene segments across various organisms including insects. A notable example is the non-coding sequences within short open reading frames (sORFs), which were once mistakenly thought to lack any functional components. Recent studies are identifying an increasing number of sORF-based AMPs. Two months ago, Marcelo and his co-workers revealed a series of AMPs from the human gut metagenome, which contains hundreds of thousands of sORFs (155). These findings suggest that these frames should become a significant identification focus in future research.

Another representative AMP using the same classic method is ranacyclin T, which was discovered by Zhang et al. through the complementary DNA (cDNA) library screening method (61). By extracting mRNA from the *Rana esculenta* frog and performing reverse transcription, Zhang et al. synthesized the cDNA and cloned it into pBlueScript vectors, which were then transformed into *E. coli* to construct cDNA library for the subsequent screening of AMP-encoding genes (61). Meanwhile, transcriptome analysis is being frequently used to identify potential AMPs. Recently, Wang et al. identified a novel AMP, CpAMP, in the Chinese horseshoe crab (160), which exhibits broad-spectrum antibacterial activity, including against multidrug-resistant strains. Thus, it has been explored for immune enhancement in animals. Besides, proteomic analyses including mass spectrometry and liquid chromatography tandem mass spectrometry are also valuable for initial screening. These approaches are frequently employed for high-throughput screening (HTS) to identify AMPs from complex protein mixtures. An example is the report from the Song group, in which 43,000 peptides were successfully identified through proteomic sequencing. Six of these peptides (P1–P4 and S1–S2) have been evaluated as potential AMPs (157).

Phage display libraries are another HTS technology used to discover functional AMPs by displaying peptide sequences on bacteriophages (e.g., M13) fused to coat proteins (e.g., pIII or pVIII). Libraries with millions to billions of peptide variants are screened against targets like bacterial membranes through biopanning. After multiple rounds of binding, washing, and amplification, high-affinity peptides are identified via sequencing. This method offers advantages such as high-throughput, vast sequence diversity exploration, and direct functional screening. For example, a cationic peptide A (KPQQHNRPLRHK) with enhanced activity against *Bacillus subtilis* was identified in this process (44). However, phage display relies on *in vivo* systems, which can face limitations like host toxicity or compatibility issues. To overcome these challenges, ribosome display has emerged as an *in vitro* alternative. This method forms peptide-ribosome-mRNA complexes, linking genotype (mRNA) and phenotype (peptide) without host cells, enabling the screening of potentially toxic peptides. Ribosome display is particularly effective for identifying AMPs targeting bacterial membranes, as it avoids host toxicity and allows the discovery of highly specific peptides. For instance, motif sequences (e.g., ALR, KVL) recognizing bacterial membranes have been identified using this approach (161). Thus, ribosome display complements phage display, offering a versatile tool for AMP discovery, especially in complex or toxic peptide interactions.

Another strategy for screening AMPs directly relies on their antimicrobial properties. In this approach, a collection of highly diverse plasmid libraries containing randomly generated open reading frames is transformed into competent cells (Fig. 2D) (162). These cells are then cultured on agar plates, followed by additional culture with specific microorganisms until bacteriostatic circles are observed. Effective AMPs are identified by isolating bacteria that exhibit clear zones of inhibition. By following this identification pathway, Michael et al. successfully identified three aminoglycoside resistance peptides, named as Arp1–3 (162).

After identifying potential AMPs through multidimensional approaches, experimental validation is essential to confirm their antimicrobial activity (8). Thus, the next step involves synthesizing these peptides, typically using SPPS, which ensures high purity and yield for subsequent testing (Fig. 2D) (163). However, SPPS can be costly and may not always produce peptides with functional conformations (164). Alternatively, plasmid-based expression in cells offers a potential solution, although further refinement is needed (162, 165). Synthesized peptides are subsequently tested using methods such as zone of inhibition assays, MIC determination, and time-kill assays to assess sterilization capabilities (Fig. 2D) (1, 166). These steps bridge discovery to application, transforming identified AMPs into validated therapeutic candidates.

Notably, regardless of the method employed, toxicity testing is essential before potential AMPs can be considered for future clinical applications. Among them, the hemolysis assay on eukaryotic cells, particularly red blood cells, is the first assay done to assess this safety. By assessing hemolytic activity, researchers can gauge the selectivity of AMPs for microbial targets versus host cells. Low hemolytic activity indicates clinical safety with minimal human cell damage. A typical example is α-hemolysin, which is produced by *S. aureus* and serves as a virulence factor due to its strong hemolytic activity on human erythrocytes (167). Another widely used method to evaluate the potential toxicity of AMPs is the *Galleria mellonella* larvae assay (168). This model is advantageous due to its simplicity, cost-effectiveness, and ethical acceptability compared to mammalian models. The larvae are injected with varying concentrations of the AMP, and survival rates are typically monitored over 24–96 hours (168). Toxicity is assessed by observing mortality, melanization, and behavioral changes such as reduced movement. By utilizing the *Galleria mellonella* larval model, Jess et al. confirmed the safety of cecropin A-melittin in the treatment of multidrug-resistant *E. coli*, indicating its potential for future applications (168).

As the AMR threat continues to escalate, the discovery of new AMPs presents a promising avenue for developing novel antimicrobial agents. By leveraging both computational and experimental approaches, researchers can explore the potential of AMPs in combating infectious diseases and enhancing human health. This multifaceted process involves a combination of bioinformatics, experimental techniques, and the exploration of natural sources (8). These technologies complement one another, enabling more effective and rapid identification and characterization of AMPs.

## CONCLUSION AND DISCUSSION

AMPs represent a promising frontier in the fight against infectious diseases and the AMR crisis. Their unique structural characteristics, excellent antimicrobial activities, and relative safety to host cells make them attractive candidates for various applications in medicine, agriculture, and food safety (8). In this review, we summarize the latest reports on novel AMPs as of the end of 2024/2025 and analyze the current landscape and trends of approved AMPs in the market. Importantly, the examples provided are not isolated instances but are interconnected by an underlying framework, forming a cohesive narrative that highlights the intrinsic relationships between different AMP applications. This approach allows us to systematically analyze the advantages and disadvantages of AMPs compared to conventional antibiotics, while also emphasizing the main methods for discovering novel AMPs in today’s rapidly evolving technological landscape. Together with a focus on groundbreaking AMPs reported in the past 5 years, this methodological discussion and application landscape constitute a core pillar of the review, offering a comprehensive and original perspective that fills a critical gap in the existing literature.

Notably, some natural bacteriocins might selectively target pathogenic bacteria while sparing the normal flora (129). Consequently, these AMPs can be optimized to function specifically within certain environments, thereby minimizing the risk of cross-reactions (129). In this context, AMPs offer significant advantages for treating infections in specific regions with unique microbiomes, such as those associated with reproductive tract infections. Modern medical studies indicate that an imbalance in the natural microbiome can lead to diseases, including secondary infections or other negative clinical consequences (169). Therefore, future research should focus on the identification of AMPs associated with this class of diseases.

## Data Availability

All data and materials supporting the findings of this study are available in the article. Additional data are available from the author upon reasonable request.
